# Predictive Application Value of Metagenomic Next-Generation Sequencing in the Resistance of Carbapenem-Resistant Enterobacteriaceae

**DOI:** 10.1155/cjid/6619016

**Published:** 2025-01-06

**Authors:** Jiacheng Tian, Chengtan Wang, Pingping Song, Zhiqing You, Xiuqin Jia, Xuan Li, Feng Pang

**Affiliations:** ^1^Department of Clinical Laboratory, Liaocheng People's Hospital, Shandong Second Medical University, Weifang, Shandong, China; ^2^Department of Clinical Laboratory, Liaocheng People's Hospital, Liaocheng 252000, Shandong, China; ^3^Christophe Mérieux Laboratory, National Institute of Pathogen Biology, Chinese Academy of Medical Sciences and Peking Union Medical College, Beijing, China; ^4^The Key Laboratory of Molecular Pharmacology, Liaocheng People's Hospital, Liaocheng 252000, Shandong, China

**Keywords:** carbapenem-resistant Enterobacteriaceae, carbapenemase, metagenomic next-generation sequencing technology, resistance genes

## Abstract

**Objective:** Although metagenomic next-generation sequencing (mNGS) technology has achieved notable outcomes in pathogen detection, there remains a gap in the research regarding its application in predicting the antibiotic resistance of pathogenic bacteria. This study aims to analyze the clinical application value of mNGS in predicting the resistance of carbapenem-resistant Enterobacteriaceae (CRE), as well as the relevant influencing factors, thereby providing valuable insights for clinical antimicrobial therapy.

**Methods:** Nonduplicate isolates of *Enterobacterales* bacteria collected from Liaocheng People's Hospital from April 2023 to June 2024 were selected, and CRE bacteria were screened. mNGS was used to detect resistance genes, and the results were compared with those of polymerase chain reaction (PCR) to evaluate the specificity and sensitivity of gene detection. Furthermore, the performance of mNGS in identifying pathogenic microorganisms and predicting antibiotic resistance was assessed by comparing the sequencing results with those of antimicrobial susceptibility testing (AST).

**Results:** A total of 46 isolates were confirmed as CRE through traditional AST and were further identified using the Vitek MS and Vitek 2 systems. The results indicated 27 isolates of *Klebsiella pneumoniae*, 14 isolates of *Escherichia coli*, 2 isolates of *Enterobacter hormaechei*, 2 isolates of *Enterobacter cloacae*, and 1 isolate of *Citrobacter freundii*. These isolates were subjected to both mNGS and PCR for detection. The calculation of the area under the receiver operating characteristic (ROC) curve demonstrated the reliability of mNGS in detecting resistance genes.

**Conclusion:** mNGS demonstrated high sensitivity in predicting the presence of carbapenemase resistance genes in CRE, showing potential in early indication of isolate resistance information, thereby facilitating timely guidance for clinical treatment strategies.

## 1. Introduction

Carbapenem-resistant Enterobacteriaceae (CRE) refer to bacteria belonging to the Enterobacteriaceae family that are resistant to at least one carbapenem antibiotic in vitro. In recent years, the resistance rate of CRE has showed an upward trend. Compared to 2015, the resistance rate of CRE detected in China increased by 60% in 2020 [1]. Consequently, how to rapidly diagnose CRE infections has emerged as a new challenge in the field of public health. The resistance mechanisms of CRE can be broadly categorized into the following: the production of carbapenemases, the loss of outer membrane porins, decreased affinity of penicillin-binding proteins to carbapenems, and the formation of bacterial biofilms. Among these, the production of carbapenemases is the most critical mechanism underlying CRE resistance, and the detection of carbapenemase production is the primary method for identifying CRE isolates.

Commonly used detection methods for carbapenemases include phenotypic detection tests, represented by the modified carbapenem inactivation method (mCIM), and molecular techniques such as polymerase chain reaction (PCR) [2]. Phenotypic tests have good sensitivity and specificity, and they are cost-effective. However, they have the disadvantages of longer detection times and the inability to detect specific genotypes; they can only determine whether carbapenemases are produced. PCR is considered the gold standard for confirming the production of carbapenemases [3], but this technique has low resolution for detecting rare carbapenemases. Early identification of pathogens and the use of appropriate antibiotics are crucial for controlling the condition of patients undergoing anti-infective therapy. However, traditional antimicrobial susceptibility testing (AST) methods are time-consuming and have low positive rates, which often delay the optimal timing of treatment. Therefore, finding more efficient detection methods is urgent.

Metagenomic next-generation sequencing (mNGS) is a new technology based on the principle of large-scale parallel sequencing. Compared with traditional diagnostic techniques, mNGS exhibits the characteristics of high sensitivity and unbiasedness. Studies have reported the application of mNGS in lower respiratory tract infections, central nervous system infections, and joint infections, indicating its potential in identifying pathogens such as *Mycoplasma*, *Chlamydia*, and *Mycobacterium tuberculosis*, which have stringent requirements for in vitro culture [4–6]. However, there is limited research on predicting pathogen resistance using mNGS. Some studies have explored the performance of mNGS in predicting the resistance of *Acinetobacter baumannii* and *Pseudomonas aeruginosa* [7, 8]. Nevertheless, the application of mNGS in predicting resistance to CRE remains largely unexplored. Therefore, we aim to validate the sensitivity and specificity of mNGS in predicting CRE resistance through AST and PCR technology and to attempt to establish a novel diagnostic procedure for detecting resistance genes.

## 2. Materials and Methods

### 2.1. Source and Identification of Isolates

The isolates were collected from nonduplicate isolates of *Enterobacterales* bacteria at Liaocheng People's Hospital from April 2023 to June 2024. All isolates were initially screened for carbapenemase activity using the CIM. Positive isolates were further identified using the Vitek MS mass spectrometer, and susceptibility testing was performed using Vitek 2 (bioMérieux, France). Ultimately, 46 isolates were confirmed as CRE. *Escherichia coli* ATCC 8739 was used for quality control of the Vitek MS identification card, while *E. coli* ATCC 25922 and *P. aeruginosa* ATCC 27853 were used for quality control of the Gram-negative bacteria susceptibility cards AST-GN13 and AST-GN16, respectively. ATCC 8739 was provided by bioMérieux, and the other standard isolates were purchased from Microbiologics, the United States of America. Control isolates were stored at −80°C for future use.

### 2.2. Identification of Carbapenemase-Encoding Genes

Genomic DNA was extracted from the studied isolates using a rapid bacterial genomic DNA isolation kit (Sangon Biotech, China). PCR was used to amplify the *bla*_*KPC*−2_, *bla*_VIM_, *bla*_IMP_, *bla*_NDM−1_, and *bla*_OXA−48_ carbapenemase genes. The primers used are listed in Table 1 [9–11]. The PCR amplification conditions were as follows: initial denaturation at 94°C for 3 min, followed by 35 cycles, each consisting of denaturation at 94°C for 1 min, annealing at 55°C for 1 min, and extension at 72°C for 2 min, with a final extension at 70°C for 5 min. After agarose gel electrophoresis of the PCR products, the bands were observed using an imaging system and compared with DNA Ladder H1 (100–1000 bp; Sangon Biotech, China; Cat# B500343) to determine their size. For positive samples, the original PCR amplification products were sent to Sangon Biotech Co., Ltd., for first-generation sequencing. The sequencing results were analyzed using BLAST to determine the genotype.

### 2.3. mNGS and Analysis

DNA was extracted from the isolates using the magnetic bead method, and the concentration of the nucleic acid to be fragmented was measured using a Qubit fluorometer. Based on the nucleic acid concentration, A (in ng/*μ*L), different volumes of nucleic acid were selected for enzymatic fragmentation: If *A* ≤ 10 ng/*μ*L, 25.0 *μ*L was directly used for the subsequent enzymatic fragmentation experiment. If 10 ng/*μ*L < *A* < 100 ng/*μ*L, 100 ng of nucleic acid was taken. The required volume was calculated, and then nuclease-free water was added to bring the total volume to 25 *μ*L. If *A* ≥ 100 ng/*μ*L, 2 *μ*L of sample was taken, and 23.0 *μ*L of nuclease-free water was added for subsequent experiments. The PMseqTM infection pathogen high-throughput detection kit (employing the combined probe anchoring sequencing method), produced by BGI Technology Co., Ltd., was used for library preparation. This process included end repair, adapter ligation, PCR amplification, library quality control, library pooling, and DNB preparation. Subsequently, metagenomic sequencing was performed using the MGI-200 platform (BGI, Shenzhen, China) with a single-end read length of 50 bp. The standard sequence files obtained after sequencing were then analyzed.

### 2.4. Statistical Analysis

The data obtained from this study were analyzed using SPSS v.26.0 software. Receiver operating characteristic (ROC) curves were constructed to evaluate sensitivity and accuracy. A *p* value < 0.05 was considered statistically significant.

## 3. Results

### 3.1. Species Confirmation and Drug Sensitivity Testing

The 46 isolates were initially subjected to carbapenemase inactivation testing, followed by identification using the Vitek MS and Vitek 2 systems, ultimately confirming them as CRE. The results indicated a 100% resistance rate to both meropenem and imipenem among all tested isolates. Specifically, among the isolates, 27 (58.7%) were identified as *Klebsiella pneumoniae*, with 5 (18.5%) isolates positive for metallo-*β*-lactamase (MBL), 20 (74.1%) positive for serine carbapenemase, and two (7.4%) negative for both MBL and serine carbapenemase. In addition, 14 (30.4%) isolates were *E. coli*, among which 13 (92.9%) were MBL-positive and one (7.1%) was serine carbapenemase-positive. Both *Enterobacter hormaechei* and *Enterobacter cloacae* were represented by two isolates each (4.3%), all of which (100%) were MBL-positive. Lastly, one isolate of *Citrobacter freundii* (100%) was MBL-positive (Table 2).

### 3.2. PCR Detection of CRE Resistance Genes

PCR and agarose gel electrophoresis were performed on the 46 confirmed CRE isolates, and the positive results were subjected to Sanger sequencing. Analysis revealed that among the 27 *K. pneumoniae* isolates, *bla*_KPC−2_, *bla*_NDM−1_, *bla*_NDM−1_+ *bla*_KPC−2_, and *bla*_IMP−1_ were detected 15, six, three, and one isolates, respectively. Two isolates were free for any carbapenemase gene. Among the 14 *E. coli* isolates, *bla*_KPC−2_ was found in one isolate and *bla*_NDM−1_ in 10 isolates. *E. hormaechei*, *E. cloacae*, and *C. freundii* isolates harbored the *bla*_NDM−1_ gene (Table S1).

### 3.3. mNGS Prediction of Antibiotic Resistance in CRE

mNGS analysis was conducted on the 46 confirmed CRE isolates. PCR and mNGS analysis results were concordant for the 27 *K. pneumoniae* isolates (*bla*_KPC−2_, *bla*_NDM−1_, *bla*_NDM−1_ + *bla*_KPC−2_, and *bla*_IMP−1_ were detected 15, 6, 3, and one isolates, respectively. Two isolates were free for any carbapenemase gene). Among the 14 *E. coli* isolates, *bla*_KPC−2_ was found in one, *bla*_OXA−48_ in one, *bla*_IMP−1_ in two, and *bla*_NDM−1_ in 10 isolates. All *E. hormaechei* and *E. cloacae* isolates harbored *bla*_NDM−1_ gene, while *C. freundii* isolate harbored both *bla*_NDM−1_ and *bla*_OXA−48_ genes (Table 3).

**Figure 1:**
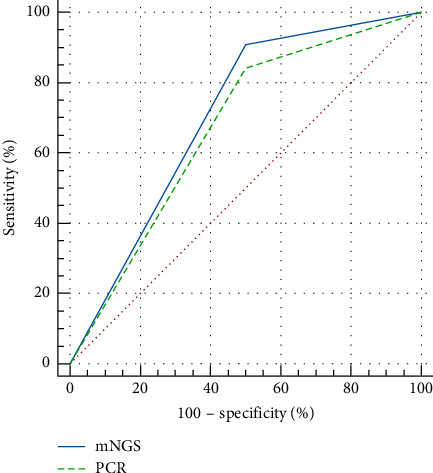
ROC curve comparing mNGS and PCR results.

Using drug sensitivity test results as the gold standard, mNGS detected 40 true positives (occurrence of *bla* gene), one true negative, one false positive, and four false negatives for resistance genes, resulting in a sensitivity of 90.91% and an accuracy of 89.13%. The ROC curve analysis was performed using MedCalc software (Figure 1), yielding an area under the curve (AUC) of 0.705 for mNGS and 0.670 for PCR, with *p* = 0.0761 > 0.05, indicating that while there was no statistically significant difference between the two methods, mNGS demonstrated a slightly higher diagnostic performance.

## 4. Discussion

*Enterobacterales* bacteria represent one of the primary pathogens responsible for hospital-acquired infections. These bacteria exhibit high resistance rates to most drugs except carbapenem antibiotics, which have thus become the primary therapeutic option. However, in recent years, the abuse of antibiotics has significantly increased carbapenem resistance among *Enterobacterales*, posing greater challenges for clinical treatment. Among the phenotypic tests for CRE detection, the modified Hodge test (MHT) has been found to accurately detect only 50% of isolates producing New Delhi MBL (NDM) [12], rendering it no longer the preferred method. The modified carbapenemase inactivation test boasts high sensitivity and specificity but, such as MHT, requires overnight incubation, which is time-consuming. While the carba NP test offers the advantages of speed and simplicity, it exhibits low sensitivity to OXA-type carbapenemases [13]. Consequently, there is a pressing need for a more rapid and comprehensive approach to detect CRE resistance. Studies have demonstrated that NGS-based AST effectively shortens the reporting time compared to traditional drug sensitivity detection techniques [8]. Nevertheless, the research on the correlation between mNGS-detected CRE resistance phenotypes and genotypes remains insufficient.

In light of this, we discussed the feasibility of using mNGS to predict CRE resistance. Isolates underwent carbapenemase inactivation tests and were identified using the Vitek MS mass spectrometer, followed by drug sensitivity testing with Vitek 2. Subsequently, mNGS was employed to determine the metagenomic data of CRE, and resistance genes were identified through software analysis, which were then compared with the resistance genes detected by PCR. Both methods yielded similar results for *bla*_KPC−2_ and *bla*_NDM−1_ detection, but mNGS demonstrated higher sensitivity in detecting *bla*_IMP−1_ and *bla*_OXA−48_ compared to PCR. By plotting ROC curves and calculating the AUC, we found that mNGS had an AUC of 0.705, while PCR had an AUC of 0.670, suggesting that as an emerging detection technology, mNGS surpasses traditional PCR methods in terms of sensitivity for resistance gene detection. PCR requires prior knowledge of the correct sequences of resistance genes to design specific primers for amplification, whereas mNGS eliminates the need for specific primer design, enabling direct parallel sequencing of the entire bacterial genome. Furthermore, we compared the predicted resistance by mNGS with the results from the Vitek system. In one case, a *K. pneumoniae* isolate tested negative for both MBL and serine-type carbapenemase by the Vitek system, yet mNGS and PCR results indicated the presence of the KPC gene, suggesting that the resistance mechanism in this isolate does not solely involve carbapenemase production. The resistance mechanisms in CRE are complex, and genetic mutations can also contribute to bacterial resistance. Our failure to observe genetic mutations may be one of the reasons for the false negatives observed in mNGS. Nonetheless, compared to traditional drug sensitivity tests or the Vitek system, mNGS has the advantage of detecting low-abundance samples without the need for microbial culture, which is crucial for early detection and monitoring of the spread of resistant bacteria.

The application of mNGS also poses certain challenges. Firstly, the cost of mNGS is relatively high compared to traditional AST methods. Secondly, the comprehensive application of mNGS is constrained by factors such as high host DNA content, nucleic acid contamination, the complexity of interpreting mNGS data, and sequencing depth. Thirdly, while a positive result from mNGS indicates the detection of pathogen nucleic acid fragments, it does not definitively confirm the expression of related products by the pathogen. However, as mNGS technology matures, it can be integrated with traditional test reports to enhance pathogen detection rates and promote the development of precision medicine concepts.

## 5. Conclusion

In this study, we have demonstrated that mNGS exhibits high sensitivity in predicting carbapenemase-producing resistance genes in CRE, suggesting its potential application in early indication of isolate resistance information. This can facilitate the prompt guidance of clinical treatment strategies.

## Figures and Tables

**Table 1:** List of utilized PCR primer sequences.

Gene	Primers (5′ to 3′)	Expected PCR product
*bla*_KPC−2_	F:ATGTCACTGTATCGCCGTCT R:TTTTCAGAGCCTTACTGCCC	893 bp
*bla*_VIM−1_	F:TTATGGAGCAGCAACGATGT R:CAAAAGTCCCGCTCCAACGA	920 bp
*bla*_VIM−2_	F:AAAGTTATGCCGCACTCACC R:TGCAACTTCATGTTATGCCG	865 bp
*bla*_IMP−1_	F:TGAGCAAGTTATCTGTATTC R:TTAGTTGCTTGGTTTTGATG	740 bp
*bla*_IMP−2_	F:GGCAGTCGCCCTAAAACAAA R:TAGTTACTTGGCTGTGATGG	737 bp
*bla*_NDM−1_	F:GGTTTGGCGATCTGGTTTTC R:CGGAATGGCTCATCACGATC	621 bp
*bla*_OXA−48_	F:TTGGTGGCATCGATTATCGG R:GAGCACTTCTTTTGTGATGGC	743 bp

**Table 2:** Phenotypic detection of carbapenemase types in the studied isolates.

Species	Enzyme			
	Metallo-*β*-lactamases positive	Serine carbapenemase positive	Both are negative	Total
*K. pneumoniae*	5	20	2	27
*E. coli*	13	1	0	14
*E. hormaechei*	2	0	0	2
*E. cloacae*	2	0	0	2
*C. freundii*	1	0	0	1
Total	23	21	2	46

**Table 3:** Comparison of mNGS and AST results.

		mNGS		Total
		Positive	Negative	
AST (gold standard)	Positive	40	4	44
	Negative	1	1	2

Total		41	5	46

## Data Availability

The data that support the findings of this study are available from the corresponding author upon reasonable request.

## Supporting Information

Table S1: Comparison of detection results across methods and specimens.
