# Supplementary material for: Predictive Application Value of Metagenomic Next-Generation Sequencing in the Resistance of Carbapenem-Resistant Enterobacteriaceae
Source: Can J Infect Dis Med Microbiol. 2025 Jan 6;2025:6619016. doi: 10.1155/cjid/6619016 (PMC11729505; doi:10.1155/cjid/6619016)
Supplement: Supporting Information — Additional supporting information can be found online in the Supporting Information section. [file 6619016.f1.docx]

Table S1| Comparison of Detection Results Across Methods and Specimens

| number | isolate | mNGS results | PCR product results | AST results | specimens |
| --- | --- | --- | --- | --- | --- |
| 1 | *E. hormaechei* | *bla_NDM-1_* | *bla_NDM-1_* | Metallo-β-lactamases positive | venous whole blood |
| 2 | *K. pneumoniae* | not detected | not detected | not detected | BALF |
| 3 | *E. cloacae* | *bla_NDM-1_* | *bla_NDM-1_* | Serine carbapenemase positive | venous whole blood |
| 4 | *K. pneumoniae* | *bla_NDM-1_*、*bla_KPC-2_* | *bla_NDM-1_*、*bla_KPC-2_* | Serine carbapenemase positive | BALF |
| 5 | *K. pneumoniae* | *bla_NDM-1_* | *bla_NDM-1_* | Metallo-β-lactamases positive | BALF |
| 6 | *K. pneumoniae* | *bla_NDM-1_* | *bla_NDM-1_* | Metallo-β-lactamases positive | BALF |
| 7 | *K. pneumoniae* | *bla_NDM-1_* | *bla_NDM-1_* | Metallo-β-lactamases positive | BALF |
| 8 | *K. pneumoniae* | *bla_KPC-2_* | *bla_KPC-2_* | Metallo-β-lactamases positive | BALF |
| 9 | *K. pneumoniae* | *bla_NDM-1_*、*bla_KPC-2_* | *bla_NDM-1_*、*bla_KPC-2_* | Serine carbapenemase positive | ascites |
| 10 | *K. pneumoniae* | not detected | not detected | Serine carbapenemase positive | sputum |
| 11 | *E. coli* | *bla_KPC-2_* | *bla_KPC-2_* | Serine carbapenemase positive | sputum |
| 12 | *E. coli* | *bla_NDM-1_* | *bla_NDM-1_* | Serine carbapenemase positive | sputum |
| 13 | *K. pneumoniae* | *bla_NDM-1_* | *bla_NDM-1_* | Metallo-β-lactamases positive | sputum |
| 14 | *K. pneumoniae* | *bla_KPC-2_* | *bla_KPC-2_* | not detected | sputum |
| 15 | *K. pneumoniae* | *bla_NDM-1_* | *bla_NDM-1_* | Metallo-β-lactamases positive | sputum |
| 16 | *E. coli* | *bla_NDM-1_* | *bla_NDM-1_* | Serine carbapenemase positive | sputum |
| 17 | *K. pneumoniae* | *bla_NDM-1_*、*bla_KPC-2_* | *bla_NDM-1_*、*bla_KPC-2_* | Metallo-β-lactamases positive | BALF |
| 18 | *K. pneumoniae* | *bla_NDM-1_* | *bla_NDM-1_* | Metallo-β-lactamases positive | BALF |
| 19 | *K. pneumoniae* | *bla_KPC-2_* | *bla_KPC-2_* | Serine carbapenemase positive | BALF |
| 20 | *E. coli* | *bla_NDM-1_* | *bla_NDM-1_* | Metallo-β-lactamases positive | ascites |
| 21 | *K. pneumoniae* | *bla_KPC-2_* | *bla_KPC-2_* | Serine carbapenemase positive | BALF |
| 22 | *K. pneumoniae* | *bla_KPC-2_* | *bla_KPC-2_* | Serine carbapenemase positive | sputum |
| 23 | *K. pneumoniae* | *bla_KPC-2_* | *bla_KPC-2_* | Serine carbapenemase positive | urine |
| 24 | *E. coli* | *bla_IMP-1_* | *bla_IMP-1_* | Metallo-β-lactamases positive | venous whole blood |
| 25 | *E. coli* | *bla_NDM-1_* | *bla_NDM-1_* | Metallo-β-lactamases positive | sputum |
| 26 | *K. pneumoniae* | *bla_KPC-2_* | *bla_KPC-2_* | Serine carbapenemase positive | sputum |
| 27 | *K. pneumoniae* | *bla_KPC-2_* | *bla_KPC-2_* | Serine carbapenemase positive | sputum |
| 28 | *K. pneumoniae* | *bla_KPC-2_* | *bla_KPC-2_* | Serine carbapenemase positive | BALF |
| 29 | *E. coli* | *bla_NDM-1_* | *bla_NDM-1_* | Metallo-β-lactamases positive | BALF |
| 30 | *E. coli* | *bla_NDM-1_* | *bla_NDM-1_* | Metallo-β-lactamases positive | BALF |
| 31 | *K. pneumoniae* | *bla_KPC-2_* | *bla_KPC-2_* | Serine carbapenemase positive | BALF |
| 32 | *E. coli* | *bla_NDM-1_* | *bla_NDM-1_* | Metallo-β-lactamases positive | sputum |
| 33 | *E. coli* | *bla_NDM-1_* | *bla_NDM-1_* | Metallo-β-lactamases positive | sputum |
| 34 | *K. pneumoniae* | *bla_KPC-2_* | *bla_KPC-2_* | Serine carbapenemase positive | sputum |
| 35 | *K. pneumoniae* | *bla_KPC-2_* | *bla_KPC-2_* | Serine carbapenemase positive | BALF |
| 36 | *C. freundii* | *bla_NDM-1_*、*bla_OXA-48_* | *bla_NDM-1_* | Metallo-β-lactamases positive | BALF |
| 37 | *K. pneumoniae* | *bla_KPC-2_* | *bla_KPC-2_* | Serine carbapenemase positive | urine |
| 38 | *E. hormaechei* | *bla_NDM-1_* | *bla_NDM-1_* | Metallo-β-lactamases positive | BALF |
| 39 | *E. cloacae* | *bla_NDM-1_* | *bla_NDM-1_* | Metallo-β-lactamases positive | BALF |
| 40 | *E. coli* | *bla_NDM-1_* | *bla_NDM-1_* | Metallo-β-lactamases positive | venous whole blood |
| 41 | *K. pneumoniae* | *bla_KPC-2_* | *bla_KPC-2_* | Serine carbapenemase positive | BALF |
| 42 | *E. coli* | *bla_IMP-1_* | not detected | Metallo-β-lactamases positive | BALF |
| 43 | *K. pneumoniae* | *bla*_IMP-1_ | *bla*_IMP-1_ | Metallo-β-lactamases positive | BALF |
| 44 | *E. coli* | *bla*_NDM-1_ | *bla*_NDM-1_ | Metallo-β-lactamases positive | BALF |
| 45 | *E. coli* | *bla*_OXA-48_ | not detected | Serine carbapenemase positive | BALF |
| 46 | *K. pneumoniae* | *bla_KPC-2_* | *bla_KPC-2_* | Serine carbapenemase positive | sputum |
